# A Cox-Based Risk Prediction Model for Early Detection of Cardiovascular Disease: Identification of Key Risk Factors for the Development of a 10-Year CVD Risk Prediction

**DOI:** 10.1155/2019/8392348

**Published:** 2019-04-09

**Authors:** Xiaona Jia, Mirza Mansoor Baig, Farhaan Mirza, Hamid GholamHosseini

**Affiliations:** School of Engineering, Computer and Mathematical Sciences, Auckland University of Technology, Private Bag 92006, Auckland 1142, New Zealand

## Abstract

Background and Objective. Current cardiovascular disease (CVD) risk models are typically based on traditional laboratory-based predictors. The objective of this research was to identify key risk factors that affect the CVD risk prediction and to develop a 10-year CVD risk prediction model using the identified risk factors. Methods. A Cox proportional hazard regression method was applied to generate the proposed risk model. We used the dataset from Framingham Original Cohort of 5079 men and women aged 30-62 years, who had no overt symptoms of CVD at the baseline; among the selected cohort 3189 had a CVD event. Results. A 10-year CVD risk model based on multiple risk factors (such as age, sex, body mass index (BMI), hypertension, systolic blood pressure (SBP), cigarettes per day, pulse rate, and diabetes) was developed in which heart rate was identified as one of the novel risk factors. The proposed model achieved a good discrimination and calibration ability with C-index (receiver operating characteristic (ROC)) being 0.71 in the validation dataset. We validated the model via statistical and empirical validation. Conclusion. The proposed CVD risk prediction model is based on standard risk factors, which could help reduce the cost and time required for conducting the clinical/laboratory tests. Healthcare providers, clinicians, and patients can use this tool to see the 10-year risk of CVD for an individual. Heart rate was incorporated as a novel predictor, which extends the predictive ability of the past existing risk equations.

## 1. Introduction

Cardiovascular disease (CVD) describes various conditions that affect the functioning of heart/cardiovascular [1]. Due to the high rate of disease morbidity, CVD has become the leading cause of mortality around the world [2–4]. In New Zealand, statistics on CVD mortality in 2017 suggests that the percentage of deaths caused by CVD is 33% [4].

Majority of cardiovascular-related deaths are premature and preventable and can be improved by effective health management by employing effective diet plans, lifestyle interventions, and drug intervention [5]. To prevent CVD, a useful approach is to assess CVD risk regularly and then introduce new lifestyle adjustments or clinical treatments accordingly.

In the past decades, a great deal of research has been done on the CVD risk estimation such as the Framingham risk scores from the Framingham Heart Study (FHS) [6, 7], the QRISK equations [8], the Europe SCORE risk equations [9], the ASSIGN scores from the Scottish Heart Health Extended Cohort (SHHEC) [10], the Prospective Cardiovascular Master (PROCAM) equations [11], and the CUORE Cohort Study formulas [12]. These CVD risk prediction models have proved their effectiveness in the health and disease management for clinicians and individuals [13–15]. The new PREDICT CVD risk assessment equation developed for primary health care among the population in New Zealand has been integrated to the electronic health records (EHRs) and a web-based software called PREDICT has been developed to support general practices manage the CVD risk in primary care [13]. The PREDICT has got 400,728 patients assessed with the CVD risk and is becoming a useful tool for decision support and health management for general practitioners.

However, challenges and issues regarding the development of CVD risk estimation models still exist. CVD risk models [16–18] are based on single risk factor which cannot realize the influence of multiple factors simultaneously. Risk models [6, 8, 19] using statistical regression methods [20–22] prefer to use classic risk factors such as age, smoking, diabetes, sex, high blood pressure, and total cholesterol to estimate the risk score. Studies [18, 19, 23–27] applying data mining or machine learning techniques for the CVD risk estimations cannot provide an absolute risk estimation, although some of these models [18, 26] tried to incorporate novel predictors in the risk models. This research aims to identify the novel risk factors for CVD detection by conventional predictors and then enhance the risk estimation by developing a multiple-variable-based risk prediction model that targets the 5-year and 10-year CVD events.

## 2. Methods

### 2.1. Study Population

The study population selected from the Framingham Original Cohort study dataset [28, 29]. We obtained the ethics approval from NHLBI [30] and the Auckland University of Technology Ethics Committee (AUTEC) (Ref: 17/385 Early Detection and Self-Management of Cardiovascular Disease Using Artificial Intelligence-Based Model). The data from this cohort study includes a total of 5079 men and women aged 30-74 years free of CVD at the baseline, of them 3189 had CVD events eventually. Details of the CVD events distribution in male and female among the study population are summarized in Table 1.

### 2.2. Data Extraction

There are 32 exams in the Framingham Original Cohort study dataset, as shown in Appendix A. Data frame collected in the first exam “Exam1” was chosen to develop the CVD prediction model because it has the maximum number of samples 5209 subjects. Data from 130 subjects were removed because of the ethics protection. The other five exams are ranging from 8 to 12, marked with italic font (as shown in Table 7 of Appendix A) and will be used for the validation for the fitted model. Data of candidate risk factors (listed in Table 2) for creating the risk model was extracted.

### 2.3. Statistical Analysis

Cox proportional hazard regression analysis [22] was selected for developing the proposed risk model (one of the most accurate method belonging to the semiparametric statistical method). This research aims to develop a prediction model using multiple parameters to estimate the probability of developing CVD for an individual. There are mainly three statistical approaches in* survival analysis*, i.e., nonparametric, semiparametric, and parametric [31]. The nonparametric approaches can only perform univariate analysis with single predictor and therefore are not suitable for the study of continuous variables [22, 32]. Both parametric and semiparametric approaches can perform multiple parameter analysis. They assume that the predictors and the log hazard rate have a linear relationship between [33]. However, the Cox proportional hazard model has an advantage that only the rank orderings of the failure and censoring times are used to estimate and test the regression coefficients [22]. The Cox model is more efficient even though the assumption of the parametric models is met. When the assumptions are not met, the Cox regression analysis can still be used efficiently with an extended Cox regression from [34], but a parametric model such as Weibull survival distribution would be a null model.

Statistical analyses were performed in R Studio platform [35]. Missing values for candidate risk factors listed in Table 2 were imputed using* Multiple Imputation* [36]. Continuous and categorical variables were transformed and imputed using algorithms modified from Maximum Generalized Variance (MGV) in the SAS PRINQUAL procedure [37]. R function* transcan* inside the “Hmisc” package was used [35].

For candidate predictors listed in Table 2, two steps of variables selection from the list were performed. The first step was conducted in a “Forward Selection” manner [38]; i.e., the univariate Cox analysis was applied to all candidate variables. Insignificant predictors were filtered out based on a significance level p value >0.05. In the second step, all selected variables from the univariate analysis were entered into the multivariate Cox regression analysis to see how the risk factors jointly impact the incidence rate for CVD. Risk factors with a p value less than 0.05 will be finally decided.

In the validation stage, two approaches were undertaken to assess the predictive ability of our fitted model, statistical validation, and empirical validation. The statistical validation was performed with respect to both discrimination and calibration. The empirical validation was defined as an empirical comparison with a general CVD risk prediction model (the Framingham office-based risk equation [6]) in a horizontal and longitudinal perspective. The horizontal comparison was conducted by comparing with the Framingham prognostic model using data collected from multiple samples at the same time point. The longitudinal comparison was conducted by comparing with the Framingham prognostic model using data collected from specific examples at different time-points (fixed time intervals follow-up) and seeing the risk trend for an individual over time.

## 3. Results

### 3.1. Derivation of a 10-Year Risk Score for CVD

Risk factors included in the risk model are age, sex, body mass index (BMI), hypertension, systolic blood pressure (SBP), cigarettes per day, pulse rate, the status of diabetes. Characteristics of risk factors were listed in Table 3. Statistics of “Min.”, “1st Qu.”, “Median”, “Mean”, “3rd Qu.”, and “Max.” of these risk factors are summarized.

The regression coefficients, hazard ratios, and their corresponding upper and lower 95% confidence intervals (CI) were estimated, as presented in Table 4. Values of the baseline hazard rate where the time point is ten years were estimated as well, shown in Table 5. The 10-year baseline hazard rate is 0.1023354 at mean values of all covariates, 0.001863652 at all covariates equal to zero. Corresponding, the survival probability (exp⁡(*basehaz*)) is 0.9027267 at mean values and 0.9981381 at all covariates equal to zero.

The Cox model has an exponential form (see Equation (1)), where t represents the time that the event occurs; *λ*(*t*) is the hazard function for a subject at time t, determined by a set of m covariates (*X*_1_, *X*_2_,…, *X*_*k*_); *β*_1_, *β*_2_,…*β*_*k*_ are the regression coefficients that measure the effect size of covariates; exp is the exponential function (exp⁡(X) = ex); *λ*_0_(*t*) is the baseline hazard rate, an arbitrary (unknown) function, corresponding to the value of the hazard when all *X*_*i*_ equal zero.

(1) $$\begin{array}{c} \lambda \left(\;t\;\right)=\lambda_{0}\left(\;t\;\right)\exp\left(\;\beta_{1}X_{1}+\beta_{2}X_{2}+\ldots +\beta_{k}X_{k}\;\right) \end{array}$$

So, the Cox model can be written as a survival function:

(2) $$\begin{array}{c} S\left(\;t\;\right)={\left[\;S_{0}\left(\;t\;\right)\;\right]}^{\exp\left(\sum_{i=1}^{k}\beta_{i}X_{i}\right)} \end{array}$$

A general formula for computing risk estimates has the following form:

(3) $$\begin{array}{c} \hat{H\left(t\right)}=1-{\left[\;S_{0}\left(\;t\;\right)\;\right]}^{\exp\left(\sum_{i=1}^{k}\beta_{i}X_{i}-\sum_{i=1}^{k}\beta_{i}{\overset{-}{X}}_{i}\right)} \end{array}$$

where H(t) is the CVD risk estimated for an individual; S0(t) is baseline survival rate at follow-up time t, where t = 10 years (see Table 5), *β*i is the regression coefficient (see Table 4), *X*_*i*_ is the value of the *i*_*th*_ risk factor (if is continuous it is the log-transformed value), ${\overset{-}{X}}_{i}$is the corresponding mean, and k denotes the number of risk factors. The CVD risk function could be derived from (3), using regression coefficients from Table 4 and the baseline hazard rates from Table 5; hence, we computed the probability of developing any type of CVD for an individual. A case of computing the absolute risk score in 10 years was demonstrated in Appendix C.

### 3.2. Nomograms

**Figure 1:**
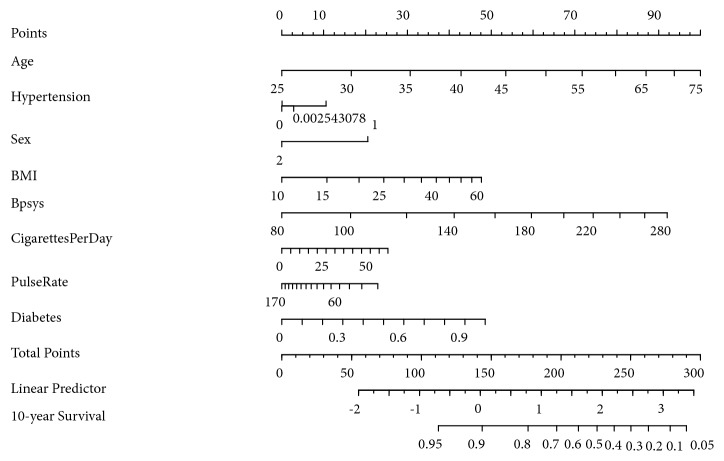
Nomogram for predicting overall survival in 10 years.

A nomogram is a two-dimensional diagram to represent a mathematical function involving several predictors [39]. It is a simple graphical illustration to approximately predict a particular event based on conventional statistical regression methods such as Cox proportional hazards model for survival analysis [40]. A nomogram is accomplishing the estimation of individual survivals in 10 years and the median survival time by years was depicted in Figure 1.

In Figure 1, each predictor has a set of n scales, and there is a mapping between each scale and the “Points” scale. The bottoms are the corresponding 10-year survival estimates, and the median survival time (years). By accumulating the total points corresponding to the specific configuration of covariates for a patient, a clinician can then manually obtain the predicted value of the event for that patient.

### 3.3. Validation

The validation of the proposed predictive risk model was performed using traditional statistics. C-index (also called receiver operating characteristic (ROC) area) [41] was used to assess the goodness of the risk model based on a bootstrap internal resampling validation. From the statistical validation analysis, we got a C-index (area under the receiver operator curve [AUROC]) of 0.71 indicating moderately good discrimination.

**Figure 2:**
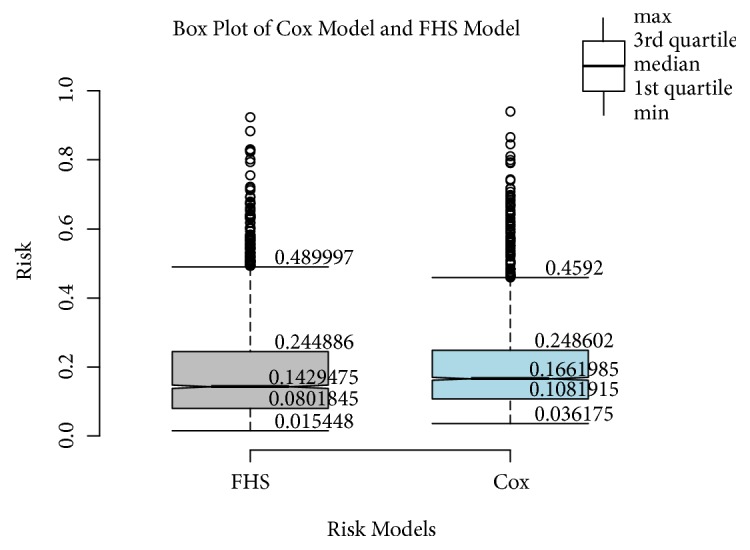
Horizontal comparison between Cox model and FHS model.

Then, we performed an empirical validation by comparing our risk model with the Framingham Heart Study model in an external dataset horizontally and longitudinally over time. In the horizontal validation process, there were 2786 samples in the external dataset, and 1693 samples have got a CVD event. Risk scores using the FHS model and the proposed risk model were computed separately. Statistics of* min (lower whisker)*,* 1st quartile (the lower hinge)*,* median*,* 3rd quartile (the upper hinge)*, and* max (the extreme of the upper whisker) *of estimated risks for all samples are depicted in Figure 2. This box-whisker graph in Figure 2 shows that the risks assessed by our Cox model are higher than the risk calculated by the Framingham model, but the error for five statistics (min, 1st Qu, median, mean, 3rd Qu., max) is within 0.02. For example, the median values of the FHS model and the Cox model are 0.1429475 and 0.1661985, respectively. For subjects with CVD event, the Cox model is much more accurate than the FHS model whereas for subjects without CVD, the Cox risk model overestimates the risk rate. Overall, the risk scale of the Cox model is consistent with the Framingham model, which highlights that the proposed Cox model is par with the FHS model.

In the longitudinal validation process, we selected four sex-specific subjects with or without CVD at the end of the Framingham Study. A summary of these four subjects is listed in Table 6 to confirm the longitudinal validation of the predicted CVD event.

**Figure 3:**
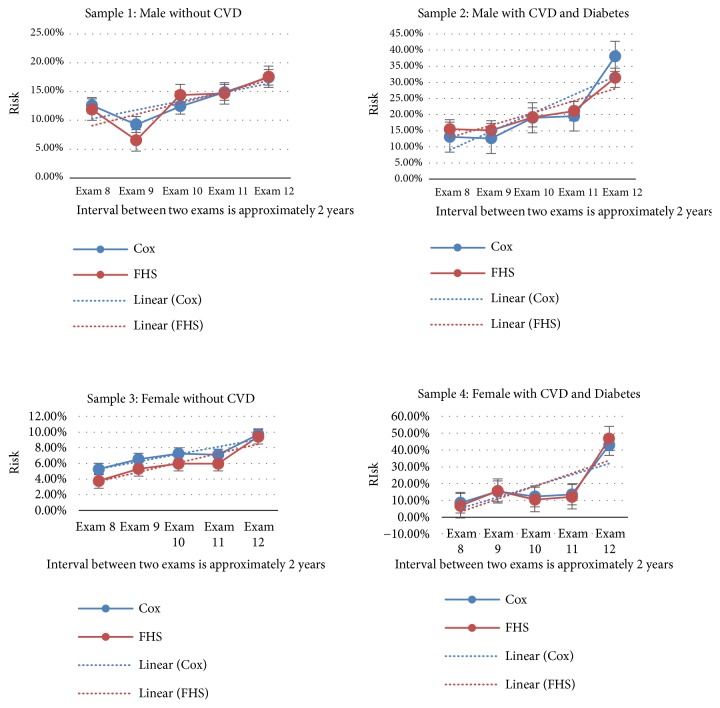
Longitudinal validation.

For each sample, data with fixed time intervals (approximately two years) from longitudinal time follow-up are extracted. The data from five exams (Exam 8, Exam 9, Exam 10, Exam 11, and Exam 12) are extracted for comparison. Data summary for sample 1, sample 2, sample 3, and sample 4 are listed in Appendix B. For each sample, the risks of developing CVD in 10 years related to the selected five exams data are separately computed using the Cox model and the Framingham model. Then the trend of risk over the years with 5% error is depicted, as shown in Figure 3. This figure shows that the trend of risks of these two models are consistent and risks for a specific sample increase over time, the dotted trend lines in each graph represent the increase in the CVD risk over time. Also, samples (both male and female) with diabetes that developed CVD will have a higher risk than the ones with no developed CVD.

## 4. Discussion

It is widely accepted that CVD has become one of the significant public health issue globally [42, 43] and contributes significantly to the annual deaths globally. Previous studies have noted the importance of identifying associated risk factors and the early detection and intervention of CVDs [44–48] and investigated reducing the risk of developing CVD in early stages. Consequently, CVD risk prediction tools based on a single variable or multiple variables have been devised to yield estimates of the CVD risk [6, 8, 9, 14, 49–51].

Motivated by the objective of early detection and risk estimation of CVD, the present study was designed to identify novel CVD risk factors, determine the effect of these factors, and then develop a risk prediction model based on the identified factors. Although risk factors could vary from one specific CVD component to another, there is sufficient evidence that different types of CVD have commonalities of risk factors. We developed and validated a 10-year risk equation for CVD risk using follow-up data rigorously measured by the Framingham Heart Study.

This investigation extends the number of risk factors by the previous general CVD risk formulations, incorporating heart rate to estimate absolute CVD risk. The approach used in this research is based on advanced statistical techniques that allow reducing the bias in the assessment of true CVD risk. The whole process of data analysis strictly follows the guideline of regression modelling strategies and survival analysis [34, 52].

We use continuous variables (age, BMI, SBP, and pulse rate) to generate the model that performs better than other similar models developed using categorical variables. Compared with simpler approaches that try to make inferences of 5-year and 10-year risk models such as the model based on logistic regression analysis [53] and the CVD risk model using Kaplan-Meier and log-rank test [46], the proposed Cox risk model is more adequate and will avoid severe errors of underestimation or overestimation [22, 34]. Moreover, this model was developed based on a more substantial number of samples and events, suggesting a valid estimation of the real risk.

### 4.1. Comparison with Other CVD Risk Prediction Tools

The old version Framingham general CVD risk function [53] is useful for identifying persons at high risk of CVD, but it was based on a limited number of risk factors (serum cholesterol, SBP, smoking history, electrocardiogram, and glucose intolerance). The new Framingham laboratory-test-based formula [6] included HDL cholesterol in the risk function. The QRISK study investigators incorporated family history as a novel risk factor by the Framingham general formulas [8]. Although researchers have published risk scores [6, 8, 53] for predicting general CVDs, these functions did not include heart rate in the risk model.

Risk models formulated by using machine learning or data mining techniques have incorporated heart rate as a risk factor but tools that can predict CVD absolute risk are fewer. For example, a prediction tool [54] focuses on the classification of CVD event by employing the ANN and the Bayesian classifier based on heart rate variability. The diagnosis CVD model [27] categorizes the CVD risk as different levels but an absolute risk score cannot be obtained. Even though a supportive tool [19] will generate the estimate of a risk score, but the user can not know how many years the score is targeting.

Some equations only focused on specific CVD outcomes. The Europe SCORE project equations were developed for the fatal cardiovascular event [9]. These risk estimation tools [7, 14, 30] are just for coronary heart disease. Also, there are some risk models aiming stroke [16, 55]. Compared with these disease-specific models to estimate the risk of developing specific CVD outcomes, the present study generated a general CVD risk tool that could predict a global CVD risk as well as the risk of developing individual components.

Moreover, compared with the laboratory-based algorithms, the present research proposed a more straightforward way to estimate 10-year CVD risk based on risk factors. An individual can assess his or her CVD risk during an office visit or his monitoring of the combination of risk factors in the risk model, either manually or use some devices like wearable sensors.

### 4.2. Implication

The CVD risk prediction model could be implemented at the primary care for population analysis and identifying the high-risk individual. This would be a transformation in healthcare management of CVD at an individual as well as at a population level. However, with a small event size of diabetes, caution must be applied to the practice of this risk model. Even though we have used multiple imputation methods to impute the missing values for diabetes, the original feature of data in-balance, which decides that the imputed data frame for the “diabetes” might still have a data in-balance there. Advanced imputation methods need to be considered in the future for avoiding unexpected outcome caused by the diabetes data in-balance.

Our research aims to provide a CVD prediction model based on key risk factors, so that it can be used at the point-of-care for better and informed decision making. Thus, risk factors based on a clinical test such as total cholesterol, HDL cholesterol were not included, but some of these risk factors have a substantial effect on the development of CVD. We have provided a valid framework for creating a risk model using the Cox regression model; future work should consider risk factors not included in our model at this moment. Thus, expanding more predictors into the risk model is an important issue for future research.

## 5. Conclusion

The proposed study devised a risk prediction model based on multivariable predictors. A novel risk factor “heart rate” was incorporated into this risk equation by conventional risk factors. A satisfying predictive ability with C-index (AUROC) of 0.71 was obtained, which ensures the accuracy of estimating risk scores. Compared with studies focusing on specific diseases, the proposed algorithm can be applied to measure the 10-year risk of CVD. Health care professionals, public health physicians, practice managers, and individuals can run the proposed model to quantify risk at a population level, during patient consultation and identify high-risk individuals for further preventive health care for the entire practice.

## Figures and Tables

**Table 1:** CVD event distribution in male and female.

	Count.	CVD Events	Age Range
Male	2294	1560	30 - 74
Female	2785	1629	30 - 74
Total	5079	3189	30 - 74

**Table 2:** Description of candidate predictors.

ORDERS	PREDICTORS	UNITS	TYPES
1	AGE	YEARS	CONTINUOUS

2	SEX	0001 MALE 0002 FEMALE	CATEGORICAL

3	BMI	KG/M2	CONTINUOUS

4	HYPERTENSION	0000 NEGATIVE 0001 TRANSIENT 0002 PERMANENT 0003 TYPE UNKNOWN 0008 DOUBTFUL	CATEGORICAL

5	HISTORY OF NERVOUS HEART	0000 NO 0001 YES, DEFINITE	CATEGORICAL

6	HISTORY OF PERICARDITIS	0000 NO 0001 YES, DEFINITE	CATEGORICAL

7	HISTORY OF OTHER CVD	0000 NO 0001 YES, DEFINITE	CATEGORICAL

8	PREMATURE BEATS	0000 NO 0001 YES, DEFINITE 0002 YES, DOUBTFUL	CATEGORICAL

9	HISTORY OF ATRIOVENTRICULAR BLOCK	0000 NO 0001 YES, DEFINITE 0002 YES, DOUBTFUL	CATEGORICAL

10	HISTORY OF RHEUMATIC FEVER	0000 NONE 0001 YES 0008 DOUBTFUL	CATEGORICAL

11	HISTORY OF ALLERGY OR ASTHMA	0000 NEGATIVE 0001 ALLERGY, ALONE 0002 BRONCHIAL ASTHMA, ALONE, 0003 ALLERGY AND ASTHMA, TOGETHER	CATEGORICAL

12	HISTORY OF THYROID DISEASE	0000 NEGATIVE 0001 HYPERTHYROID ONLY 0002 HYPOTHYROID ONLY	CATEGORICAL

13	HISTORY OF SUBACUTE ENDOCARDITIS	0000 NO 0001 YES	CATEGORICAL

14	BLOOD PRESSURE SYSTOLIC	MM HG	CONTINUOUS

15	BLOOD PRESSURE DIASTOLIC	MM HG	CONTINUOUS

16	CIGARETTES PER DAY	LAPSE, FORM 8/50	CONTINUOUS

17	CIGARS PER DAY	LAPSE, FORM 8/50	CONTINUOUS

18	PIPERS PER DAY	LAPSE, FORM 8/50	CONTINUOUS

19	PULSE RATE	PER MINUTE	CONTINUOUS

20	DIABETES	0000 NO 0001 YES, DEFINITE	CATEGORICAL

**Table 3:** Summary statistics for risk factors used in risk model.

Predictors	Variables	Min.	1st Qu.	Median	Mean	3rd Qu.	Max.
AGE	Age	28	37	44	44.15	51	74
SEX	Sex	1	1	2	1.548	2	2
BMI	Bmi	14.12	22.66	25.17	25.61	27.92	56.68
HYPERTENSION	Hyp	0	0	0	0.147	0	1
BLOOD PRESSURE SYSTOLIC	Bps	84	122	136	138.6	150	270
CIGARETTES PER DAY	Cgrpd	0	5	20	16.26	20	60
PULSE RATE	Pr	37	67	75	75.61	83	170
DIABETES	Dia	0	0	0	0.0197	0	1

**Table 4:** Regression coefficients and hazard ratios in risk model.

Predictors	Variables	coef*∗*	Hazard Ratio	lower .95	upper .95
AGE	log of age	2.083643	8.033686	6.4082	10.0716
SEX	sex	-0.469719	0.625178	0.5787	0.6754
BMI	log of bmi	0.608864	1.838342	1.4368	2.3521
HYPERTENSION	hyp	0.241461	1.273108	1.1342	1.429
BLOOD PRESSURE SYSTOLIC	log of bps	1.682571	5.37937	3.7938	7.6277
CIGARETTES PER DAY	cgrpd	0.009669	1.009716	1.0065	1.013
PULSE RATE	log of pr	-0.30209	0.739271	0.5879	0.9297
DIABETES	dia	1.087501	2.96685	2.3244	3.7869

*∗* Estimated regression coefficient.

**Table 5:** Baseline hazard and survival at 10 years.

	Covariates at mean value	Covariates equal to zero
Baseline hazard estimate	0.1023354	0.001863652
Baseline survival estimate	0.9027267	0.9981381

**Table 6:** Data summary for samples in the longitudinal validation.

Samples	Gender	CVD	Diabetes
Sample 1	Male	*✘*	*✘*
Sample 2	Male	✓	✓
Sample 3	Female	*✘*	*✘*
Sample 4	Female	✓	✓

**Table 7:** Exams in the Framingham Original Cohort study data set.

Exams	Exam Date Range	Age Range	Mean Age	Attendees
Exam 1	1948 - 1953	28 - 74	44	5209
Exam 2	1950 - 1955	31 - 65	46	4792
Exam 3	1952 - 1956	32 - 67	48	4416
Exam 4	1954 - 1958	34 - 69	50	4541
Exam 5	1956 - 1960	37 - 70	52	4421
Exam 6	1958 - 1963	38 - 72	54	4259
Exam 7	1960 - 1964	40 - 74	55	4191
* Exam 8 *	*1962 - 1966*	*42 - 76*	*57*	*4030*
* Exam 9 *	*1964 - 1968*	*44 - 78*	*59*	*3833*
* Exam 10 *	*1966 - 1970*	*46 - 80*	*61*	*3595*
* Exam 11 *	*1968 - 1971*	*49 - 81*	*62*	*2955*
* Exam 12 *	*1971 - 1974*	*50 - 83*	*64*	*3261*
* Exam 13 *	*1972 - 1976*	*53 - 85*	*66*	*3133*
Exam 14	1975 - 1978	55 - 88	68	2871
Exam 15	1977 - 1979	57 - 89	69	2632
Exam 16	1979 - 1982	59 - 91	70	2351
Exam 17	1981 - 1984	61 - 93	72	2179
Exam 18	1983 - 1985	63 - 94	74	1825
Exam 19	1985 - 1988	65 - 96	75	1541
Exam 20	1986 - 1990	67 - 97	77	1401
Exam 21	1988 - 1992	69 - 99	79	1319
Exam 22	1990 - 1994	72 - 101	80	1166
Exam 23	1992 - 1996	73 - 101	81	1026
Exam 24	1995 - 1998	76 - 103	83	831
Exam 25	1997 - 1999	78 - 104	84	703
Exam 26	1999 - 2001	79 - 103	86	558
Exam 27	2002 - 2003	82 - 104	87	414
Exam 28	2004 - 2005	84 - 104	89	303
Exam 29	2006 - 2007	85 - 102	91	218
Exam 30	2008 - 2010	88 - 102	92	141
Exam 31	2010 - 2011	90 - 99	92	91
Exam 32	2012 - 2014	93 - 106	96	40

**Table 8:** Exam data for Sample 1: male without CVD.

Exams	age	bmi	bps	pr	cgrpd	trt	hyp	dia	smk
Exam 8	44	26.386894	120	82	40	0	0	0	1
Exam 9	45	26.826676	120	80	0	0	0	0	0
Exam 10	47	27.467643	118	70	20	0	0	0	1
Exam 11	49	28.222249	110	76	44	0	0	0	1
Exam 12	52	28.675012	110	80	50	0	0	0	1

**Table 9:** Exam data for Sample 2: male with CVD and diabetes.

Exams	age	bmi	bps	pr	cgrpd	trt	hyp	dia	smk
Exam 8	45	27.74258	132	83	20	0	0	0	1
Exam 9	47	26.26118	124	80	20	0	0	0	1
Exam 10	49	27.664352	130	78	20	0	1	0	1
Exam 11	51	27.121914	130	90	20	0	1	0	1
Exam 12	53	24.816551	122	82	20	0	0	1	1

**Table 10:** Exam data for Sample 3: female without CVD.

Exams	age	bmi	bps	pr	cgrpd	trt	hyp	dia	smk
Exam 8	44	20.776333	110	70	20	0	0	0	1
Exam 9	46	20.265439	120	70	20	0	0	0	1
Exam 10	48	22.312012	118	73	20	0	0	0	1
Exam 11	50	21.797119	114	82	20	0	0	0	1
Exam 12	52	21.797119	130	76	20	0	0	0	1

**Table 11:** Exam data for Sample 4: female with CVD and diabetes.

Exams	age	bmi	bps	pr	cgrpd	trt	hyp	dia	smk
Exam 8	46	21.793044	130	65	3	0	1	0	1
Exam 9	48	21.967388	170	75	16	0	1	0	1
Exam 10	50	22.494583	140	60	8	0	1	0	1
Exam 11	53	22.31746	140	63	8	0	1	0	1
Exam 12	54	23.380197	160	58	2	1	1	1	1

**Table 12:** Data summary for the subject 15018644.

PREDICTORS	VALUES	UNITS
AGE	44	YEARS
SEX	1	MALE
BMI	26.38689413	KG/M2
HYPERTENSION	0	NO
TREATMENT OF HYPERTENSION	0	NO
BLOOD PRESSURE SYSTOLIC	120	MM HG
CIGARETTES PER DAY	40	LAPSE
SMOKING	1	YES
PULSE RATE	82	PER MINUTE
DIABETES	0	NO
*COX MODEL RISK*	*12.57*%	
*FHS MODEL RISK*	*11.86*%	

## Data Availability

The cardiovascular disease (CVD) data used to support the findings of this study were supplied by Framingham Heart Study-Cohort (FHS-Cohort) under license and so cannot be made freely available. Requests for access to these data should be made with Open BioLINCC Studies Group through this website https://biolincc.nhlbi.nih.gov/studies/framcohort/.

## Appendix

### A. Exams in the Framingham Original Cohort Study Dataset

See Table 7.

### B. Data Summary for Samples

See Tables 8–11.

### C. Computation of Absolute Risk

Here, we take a specific subject to illustrate the process of risk score calculation. This sample is a 44-year-old man not having diabetes and hypertension. He has a systolic blood pressure of 120 mm Hg, pulse rate of 82 per minute, BMI of 26.38689413 kg/*m*_2_ and is a current smoker smoking 40 lapses per day, as shown in Table 12.

The risk estimate based on the Cox model is calculated as follows:

(C.1) ∑i=1kβiXi2.083643∗log⁡44−0.469719∗1+0.608864∗log⁡26.386894+0.241461∗0+1.682571∗log⁡120−0.302090∗log⁡82+0.009669∗40+1.087501∗0=16.518741

(C.2) ∑i=1kβiX−i2.083643∗3.768−0.469719∗1.548+0.608864∗3.230+0.241461∗0.1469+1.682571∗4.913−0.302090∗4.311+0.009669∗13.96+1.087501∗0.02001=16.518741

(C.3) Ht^1−S0texp⁡∑i=1kβiXi−∑i=1kβiX−i=1−0.9027267exp⁡16518741−16.247045=0.125658≈12.57%

## Additional Points

The main contribution of the present study is developing a risk prediction model for early detection of CVD. More specifically, the contribution can be summarized in four major respects: firstly, a novel risk factor “heart rate” was identified as significant for the development of CVD; secondly, an CVD risk prediction model aiming for early detection of CVD was developed based on various risk factors; thirdly, an absolute risk score in 10 years of CVD can be calculated using this risk model; lastly, multiple forms of the risk estimation of CVD, namely risk equation and nomogram, were also developed.
